# Cerebellar Dysfunction in Multiple Sclerosis

**DOI:** 10.3389/fneur.2017.00312

**Published:** 2017-06-28

**Authors:** Alastair Wilkins

**Affiliations:** ^1^MS and Stem Cell Group, University of Bristol, Learning and Research, Southmead Hospital, Bristol, United Kingdom

**Keywords:** multiple sclerosis, demyelinating diseases, ataxia, cerebellar diseases, purkinje cells

## Abstract

Multiple sclerosis (MS) commonly affects the cerebellum causing acute and chronic symptoms. Cerebellar signs contribute significantly to clinical disability, and symptoms such as tremor, ataxia, and dysarthria are particularly difficult to treat. Increasing knowledge concerning the pathophysiology of cerebellar disease in MS from human postmortem studies, experimental models, and clinical trials has raised the hope that cerebellar symptoms will be better treated in the future.

## Introduction to Multiple Sclerosis (MS)

Multiple sclerosis is an inflammatory disease of the central nervous system of unknown etiology. Typically patients have an initial relapsing and remitting course [relapsing remitting MS (RRMS)] followed by, in the majority of case, secondary progressive MS (SPMS) during which patients develop slow, insidious accumulation of disability (1). A small percentage of patients with MS have progressive disability from onset [primary progressive MS (PPMS)]. Despite extensive research and increasing knowledge of pathophysiological mechanisms, there is still no cure for the disease. Significant advances in therapeutics have occurred, and novel therapies, including natalizumab, alemtuzumab, fingolimod, and dimethyl fumarate have important disease-modifying effects (2).

The cerebellum and its efferent and afferent pathways are commonly affected in MS; and cerebellar ataxia is a common symptom of the disease, particularly in progressive disease (3, 4). Despite affecting the entire central nervous system, there are aspects of cerebellar involvement in MS that warrant specific attention and may give important insights into potential mechanisms and treatments for progressive disease. Within this review clinical aspects, pathological changes, monitoring of cerebellar changes in MS, and treatments for cerebellar disease will be discussed. In addition, various experimental models of cerebellar inflammation will be reviewed and how they may inform on future potential therapies.

## Clinical Features of Cerebellar Dysfunction in MS

Coordination problems are common in MS and occur predominantly due to pathology within the cerebellum itself or impairment in cerebellar connections, including proprioceptive afferent inputs. Patients with MS may present with either acute cerebellar dysfunction relating to acute relapse or chronic cerebellar problems in progressive disease. Cerebellar pathology may lead to limb, gait, and truncal ataxia, dependent on precise lesional site, as well as other cerebellar features such as gaze-evoked nystagmus, dysarthria, and tremor.

Involvement of the cerebellum and brainstem connections occurs during MS relapse not infrequently. Cerebellar relapse at disease onset also seems to be associated with increased risk of cerebellar involvement during subsequent relapse (5). Multivariate analyses have suggested that involvement of the cerebellum at onset of the disease is associated with worse prognosis [shorter time to expanded disability status scale (EDSS) score 6] (6). In a recent database study of approximately 15,000 patients who experienced a total of nearly 50,000 relapses, cerebellar relapses accounted for approximately 10% of all relapses; being more frequent in men and in those with longer duration of disease (7). Cerebellar/brainstem relapses are also associated with poor relapse recovery which, in itself, is associated with earlier onset of progressive disease (8).

In patients with established MS, ataxia is thought to occur in about 80%, with symptoms particularly prevalent in those with progressive disease (3, 4). MS tremor is thought to arise predominantly as a consequence of cerebellar and/or thalamic disease (9). Tremor may affect limbs, trunk, vocal cords, and head (titubation). Intention and postural tremors are the commonest types, although rest and rubral tremors occur rarely (10, 11). Although significantly disabling, severe tremor is a relatively rare consequence of MS, occurring in 3% of patients in one study (11). Severe tremor in this study was associated with indicators of global disability, such as high EDSS. The pathophysiology of MS tremor is complex and likely to involve cerebellar connections, as well as cortical and basal ganglia connections. A pure cerebellar syndrome (in the absence of involvement of other parts of the central nervous system) should warrant the search for an alternative explanation, such as a metabolic disorder or an inherited cerebellar disorder. Gait ataxia is thought to arise predominantly due to damage to the anterior lobe of the cerebellum (12). Cerebellar dysarthria appears uncommonly at disease onset but is common in those with advanced secondary progressive disease. Paroxysmal symptoms of MS are relatively infrequent, and paroxysmal dysarthria with ataxia has been reported in MS and is thought to occur due to midbrain pathology (13, 14).

The intriguing role of the cerebellum in cognitive processing is a subject in intense research, and there is some suggestion that involvement of the cerebellum may be linked to defects in cognitive processes in MS (15). Injury to the cerebellum (from whatever cause) has been linked to deficits in verbal fluency, working memory, and attention, as well as executive dysfunction (16). Interestingly, the cognitive profile of MS patients with cerebellar lesions differs to those without cerebellar involvement (17). Reduced total cerebellar volume scores on magnetic resonance imaging (MRI) are associated with worse performance on cognitive tests (15). Specifically, volume loss of the posterior–inferior cerebellum is associated with poor cognitive function, whereas volume loss to anterior cerebellum is associated with motor dysfunction (12).

In summary, cerebellar dysfunction is a feature of both RRMS and progressive MS leading to a range of neurological manifestations. In general, involvement of the cerebellum is linked to increased disability and worse prognosis, which, given the important role of cerebellar connections in motor control, is perhaps not surprising.

## Cerebellar Pathology in MS

**Figure 1:**
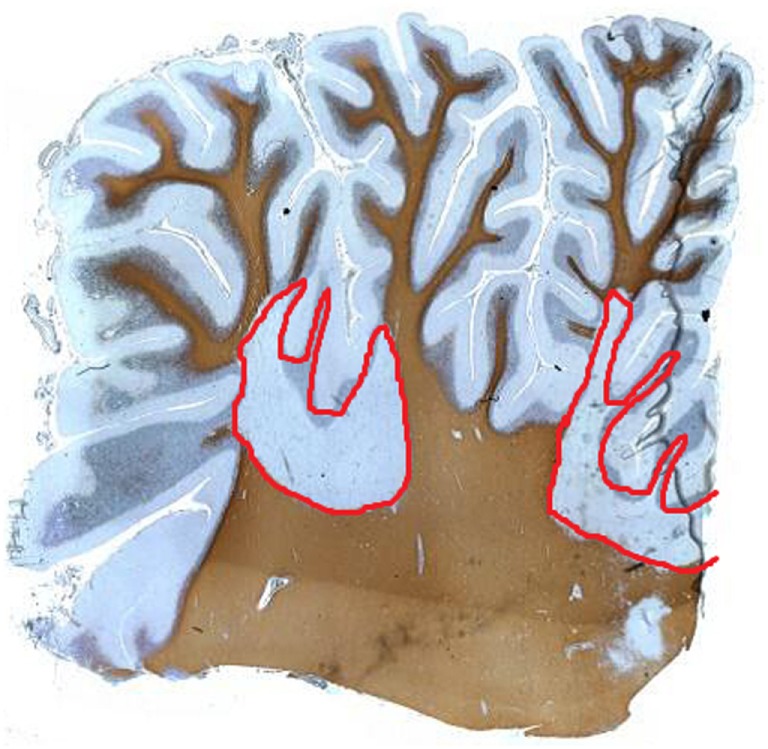
Proteolipid protein staining of human cerebellum of a patient with multiple sclerosis showing extensive white matter demyelination (red), which extends into the gray matter.

Cerebellar white matter lesions are well described in the literature and often apparent on MRI scans of patients with MS. Cerebellar peduncles are common lesional sites. Demyelination commonly occurs in cerebellar white matter (Figure 1). In addition, observations concerning gray matter demyelination in cerebral cortex has led to studies evaluating gray matter disease in the cerebellum (18, 19). Indeed, the cerebellar cortex appears a major site for demyelination with one study reporting 38.7% of the cerebellar cortex being affected in a cohort of PPMS and SPMS patients (20). Interestingly, in this study, the majority of gray matter lesions appeared independent of white matter lesions, with some tissue blocks showing very extensive gray matter demyelination in the near absence of underlying white matter disease. Cerebellar cortical lesions are typically classified as leukocortical (extending from white matter into adjacent gray matter); intracortical (purely gray matter) arising around inflamed veins and venules; and subpial lesions that are “band-like” and parallel to the meningeal surface (20). The latter are abundant in patients with progressive MS. Observations concerning the relationship of subpial demyelination and meningeal inflammation have been suggested a possible driver for neurodegenerative processes in progressive MS, although causative proof is needed (21).

Neuronal pathology in the MS cerebellum is less well defined. The study by Kutzelnigg et al. (mentioned above) showed neuronal pathology with some reductions in Purkinje cell density in lesions (compared to control) (20). No significant reductions in Purkinje cell densities were seen in non-lesional cerebellar gray matter. Our own study confirmed these observations and also showed changes in neurofilament phosphorylation states in Purkinje cells (22). We noted abnormal neurofilament phosphorylation with loss of dephosphorylated neurofilaments and increased expression of hyperphosphorylated neurofilaments. In addition, axonal spheroids (representing transection of the Purkinje cell axon) were note within leukocortical lesions, indicating significant Purkinje cell pathology within the MS cerebellum. Alterations in neurofilament phosphorylation states and axonal transection are reported within cerebral white matter (23). In a further study of MS cerebellum, we showed reductions in neuronal and myelin markers with evidence for increased lipid peroxidation end products (end products of oxidative injury to polyunsaturated fatty acids found in cell membranes) (24). We also found elevation in superoxide dismutase enzyme expression in MS cerebellum (compared to control) but a relative lack of other antioxidant enzyme expression, suggesting a possible mechanism for the extensive oxidative stress-related injury seen.

Other changes in Purkinje cell phenotype have been documented in MS, notably changes in ion channel expression and receptor profiles. The Na_v_1.8 sensory neuron-specific sodium channel is normally expressed at very low levels in Purkinje cells, but its expression is markedly upregulated in MS (25). In addition, annexin light chain (p11) that facilitates the functional expression of this ion channel is also upregulated in Purkinje cells (26). In experimental models, aberrant expression of Na_v_1.8 in Purkinje cells causes significant abnormalities of firing patterns of these cells (27, 28).

The interesting phenomenon of Purkinje cell heterokaryon formation has been noted to occur in MS cerebellum (29). This process appears to occur almost exclusively in Purkinje cells within the central nervous system and denotes the appearance of binucleated cells. Binucleation is thought to occur by cell fusion, a process by which the nucleus of a non-Purkinje cell is donated and integrates with the Purkinje cell (30). The commonest type of “donor” cell in experimental paradigms of cell fusion are bone marrow-derived cells, and researchers have postulated the donation of a foreign nucleus to the Purkinje cell may be a mechanism of protecting the cell from injury, since expression of donor cell genes occurs. In MS, binucleated Purkinje cells are significantly increased compared to control, suggesting a possible adaptive process occurring in response to the disease.

There still remains much to be understood about the pathology of the cerebellum in MS; for instance, there is a paucity of information on processes occurring in the molecular or granular layers; and histopathological correlates of cerebellar symptoms are lacking. Many of the pathological findings in MS cerebellum are shared with other neurological conditions affecting the cerebellum (e.g., neurofilament changes; Purkinje cell injury). Understanding which pathological features are specific to MS may help design future therapies. Furthermore, understanding why cerebellar pathology is linked to a worse prognosis in the disease may help design therapies for cerebellar dysfunction in MS.

## Monitoring Cerebellar Disease in MS

The major disability rating scales used in MS incorporate estimations of ataxia to a variable degree. These scales are used to provide a semiquantitative description of disability in the disease and remain prominent outcome measures of new therapies in clinical trials. The EDSS is the most commonly used disability scale in MS trials, and its quantification of disability relies heavily on motor dysfunction (31). As part of the grading, disturbances of functional systems (FS), such as visual function or bowel and bladder function, are assessed and can contribute to the overall score attributed. The cerebellar system is one of the FS tested, and increasing levels of ataxia may contribute to the overall EDSS level. Other scales also incorporate cerebellar function. For instance, the multiple sclerosis functional composite has a 9-hole peg test as one of the three tests involved, which relies heavily on the integrity of cerebellar functioning (32). The relevance of specific ataxia ratings scales has not been studied in MS in detail. Primarily these scales have been developed for assessment of “pure” cerebellar or spinocerebellar syndromes, typically the inherited ataxias.

Magnetic resonance imaging studies have confirmed extensive cerebellar involvement in both RRMS and progressive MS (33, 34). The revised McDonald criteria for the diagnosis of MS recognize infratentorial regions of the central nervous system to be a typical lesional site in MS (35). Recent diffusion-weighted MRI (DWI) techniques using a diffusion tensor model have changes in white matter tracts in MS, notably cerebellar peduncles. One study revealed abnormalities in tractography signals within the superior cerebellar peduncle, which correlated with upper limb dysfunction in patients with PPMS (36). Indeed, the presence of T2 lesions within cerebellar peduncles on MRI was associated with cerebellar and ambulatory symptoms in a large imaging study (37). As stated above, cerebellar gray matter disease may be extensive in pathological samples. A combined MRI and posturography study showed that gray matter atrophy of the superior lobules of the cerebellum (IV, V, VI), and lobules VIII correlated with worse posturometric values (38). Magnetic resonance spectroscopy (MRS) has been used to study neuronal markers, typically *N*-acetyl aspartate, in patients with MS looking for evidence of neuronal/axonal loss in progressive disease. Persistent cerebellar dysfunction has been linked with MRS markers of axonal loss in the cerebellum of patients with progressive MS (39).

Other cerebellar monitoring techniques, such as saccadic movements using eye-tracking technology, may become useful methods of monitoring cerebellar disease in MS (40). Defining suitable measures of cerebellar disease burden may be of importance in the future should disease modifying or symptomatic therapies be developed for specific cerebellar issues related to MS.

## Experimental Models of Cerebellar Inflammation and Neurodegeneration

The most well-established animal model for MS is experimental autoimmune encephalomyelitis (EAE). It is, however, not without its limitations, since many of the features of MS fail to be replicated in the model. Cerebellar inflammation occurs commonly during the course of EAE. The specific mode of inducing EAE may influence the predilection for cerebellar involvement (41). Purkinje cell dysfunction has been show to occur in EAE with reduced synaptic function (42). Loss of Purkinje cell and gray matter pathology have also been documented in EAE (43). Several models of dysmyelination and non-immune myelin loss have been studied. A model of slow, progressive myelin loss in a naturally occurring rat mutant is associated with extensive axonal changes (spheroid formation and neurofilament dephosphorylation) within the cerebellum (44).

Ion channel abnormalities in EAE have been noted for some time and form the basis for ongoing trials of sodium channel blockers in MS. Persistent sodium ion influx into the axon (associated with failure of ATP-dependent sodium/potassium exchange mechanisms in the context of reduced energy availability) leads to reversal of the sodium/calcium exchanger and thus excess intra-axonal calcium ions accumulate. Sodium channel blockade certainly appears to ameliorate axonal injury in EAE, but so far human trials have been less impressive (45). Indeed, ion channel abnormalities in Purkinje cells of mice affected by EAE have been documented, with aberrant expression of Na_v_1.8 channels causing alteration of electrophysiological properties of Purkinje cells (28).

The finding of cell fusion in MS cerebellum noted above has been postulated as a potential mechanism for Purkinje cell rescue in various neurological diseases. Transplantation of human mesenchymal stem cells into mice which have subsequently been given EAE leads to expression of human markers within the cerebellum of the these mice, suggesting that in EAE cell fusion occurs (46). Whether this leads to some form of neuroprotection in the cerebellum is unclear.

## Treatments for Cerebellar Disease in MS

Treatments for MS fall into the categories of symptomatic treatments for established symptoms and disease-modifying therapies (DMTs), which aim to reduce the burden of disease. The design of DMT trials has not sought to determine whether these drugs improve cerebellar function specifically. The majority of trials have used reduction in annualized relapse rates and EDSS scores (as a marker of disability progression). As mentioned earlier, the EDSS does have cerebellar function as part of the assessment, but its utility in picking out specific effects of drugs on cerebellar function is limited. However, cerebellar relapses are associated with an increased risk of disability accrual (relative to some other relapse types, such as sensory) (47). Thus, reducing relapses by DMTs is likely to reduce the burden of cerebellar disease in the long term. Interestingly, analysis of an alemtuzumab trial (CAMSS223 versus beta-interferon) demonstrated improvements in the cerebellar FS score of the EDSS (48).

Non-pharmacological approaches to MS ataxia are commonly employed, of which physiotherapy regimens are the most widely accepted. Balance-specific exercises involving somatosensory and motor strategy facilitation are generally employed to varying degrees (49). Improving core stability in patients with balance problems may be effective, and lumbar stabilization exercises (which improve core trunk muscles, leading to effects on postural control, ambulation, and skilled motor function) are often incorporated into MS rehabilitation programs (50). In addition, task-oriented training enhances ambulation and postural control in MS patients due to the promotion of motor learning (51). In general, combinations of these physiotherapy approaches are thought to be most beneficial (52).

Pharmacological approaches to improving ataxic symptoms are generally disappointing, and newer therapies are needed. The most recent Cochrane review of treatments for ataxia in MS (which reviewed six randomized placebo-controlled trials) concluded that absolute and comparative efficacy and tolerability of pharmacotherapies are poorly documented and no recommendations could be made (53). Small open label studies or case reports have suggested benefits for a range of drugs for the treatment of tremor. Isoniazid, propranolol, and levetiracetam have been studied, although the data on their use are not convincing (the number of patients involved in these trials was generally very low, and limited conclusions could be drawn) and they are not widely used (54–59). Several randomized controlled trials of cannabis extracts have concluded that cannabinoids appear to have no beneficial effect on MS tremor (60–62). Paroxysmal ataxia and dysarthria have been reported in MS, albeit rarely, and there is some suggestion that they may respond to carbamazepine, in a similar way to other paroxysmal symptoms of MS, such as tonic spasm (63).

The majority of reports of stereotactic surgery in MS tremor have targeted thalamic structures with variable results (64, 65). In the Cochrane review, one neurosurgical study of thalamotomy versus thalamic stimulation was included (53). Tremor was abolished by both thalamotomy and thalamic stimulation in all patients immediately postsurgery (66). However, tremor returned in almost all MS patients after 6 months (albeit of less severity than preoperative levels) and general disability scores were unchanged. The short-lived nature of the response is seen in other studies of surgical treatments of MS tremor (67). Improvement in quality of life measures following thalamic stimulation, including improvement in ability to feed oneself, has been demonstrated in selected cases (68).

There is a great need to improve therapeutic options for symptomatic treatments of cerebellar symptoms and to provide neuroprotection within the cerebellum. The design of trials aimed specifically at cerebellar protection in MS will be challenging due to the paucity of good outcome measures, although improvements in imaging techniques will help. Refinements in neurosurgical techniques may help patients with severe ataxic tremors. Increased understanding of the pathophysiology of cerebellar disease in MS will aid the search for new drug therapies. A number of trials are now being pioneered specifically aimed at slowing disease progression. Newer therapies, such a stem cell therapies, are being developed. The observation of Purkinje cell fusion as a potential neurorestorative mechanism makes the prospect of stem cell treatments for MS cerebellar disease particularly attractive.

## Author Contributions

The author confirms being the sole contributor of this work and approved it for publication.

## Conflict of Interest Statement

The author declares that the research was conducted in the absence of any commercial or financial relationships that could be construed as a potential conflict of interest.

## References

[1] Lublin FD Reingold SC Cohen JA Cutter GR Sørensen PS Thompson AJ Defining the clinical course of multiple sclerosis: the 2013 revisions. Neurology (2014) 83(3):278–86. 10.1212/WNL.0000000000000560 24871874 PMC4117366

[2] Kalincik T Brown JWL Robertson N Willis M Scolding N Rice CM Treatment effectiveness of alemtuzumab compared with natalizumab, fingolimod, and interferon beta in relapsing-remitting multiple sclerosis: a cohort study. Lancet Neurol (2017) 16(4):271–81. 10.1016/S1474-4422(17)30007-8 28209331

[3] Kurtzke JF Beebe GW Nagler B Nefzger MD Auth TL Kurland LT Studies on the natural history of multiple sclerosis. V. Long-term survival in young men. Arch Neurol (1970) 22(3):215–25. 10.1001/archneur.1970.00480210025003 5411678

[4] Swingler RJ Compston DA. The morbidity of multiple sclerosis. Q J Med (1992) 83(300):325–37. 1631264

[5] Mowry EM Deen S Malikova I Pelletier J Bacchetti P Waubant E. The onset location of multiple sclerosis predicts the location of subsequent relapses. J Neurol Neurosurg Psychiatry (2009) 80(4):400–3. 10.1136/jnnp.2008.157305 19066192

[6] Weinshenker BG Rice GP Noseworthy JH Carriere W Baskerville J Ebers GC. The natural history of multiple sclerosis: a geographically based study. 3. Multivariate analysis of predictive factors and models of outcome. Brain (1991) 114(Pt 2):1045–56. 10.1093/brain/114.2.1057 2043940

[7] Kalincik T Buzzard K Jokubaitis V Trojano M Duquette P Izquierdo G Risk of relapse phenotype recurrence in multiple sclerosis. Mult Scler (2014) 20(11):1511–22. 10.1177/1352458514528762 24777276

[8] Novotna M Paz Soldán MM Abou Zeid N Kale N Tutuncu M Crusan DJ Poor early relapse recovery affects onset of progressive disease course in multiple sclerosis. Neurology (2015) 85(8):722–9. 10.1212/WNL.0000000000001856 26208962 PMC4553030

[9] Koch M Mostert J Heersema D De Keyser J. Tremor in multiple sclerosis. J Neurol (2007) 254(2):133–45. 10.1007/s00415-006-0296-7 17318714 PMC1915650

[10] Alusi SH Worthington J Glickman S Bain PG. A study of tremor in multiple sclerosis. Brain (2001) 124(Pt 4):720–30. 10.1093/brain/124.4.720 11287372

[11] Pittock SJ McClelland RL Mayr WT Rodriguez M Matsumoto JY. Prevalence of tremor in multiple sclerosis and associated disability in the Olmsted County population. Mov Disord (2004) 19(12):1482–5. 10.1002/mds.20227 15390075

[12] D’Ambrosio A Pagani E Riccitelli GC Colombo B Rodegher M Falini A Cerebellar contribution to motor and cognitive performance in multiple sclerosis: an MRI sub-regional volumetric analysis. Mult Scler (2016). 10.1177/1352458516674567 27760859

[13] Blanco Y Compta Y Graus F Saiz A. Midbrain lesions and paroxysmal dysarthria in multiple sclerosis. Mult Scler (2008) 14(5):694–7. 10.1177/1352458507087846 18566032

[14] Valentino P Nisticò R Pirritano D Bilotti G Del Giudice F Sturniolo M Lamotrigine therapy for paroxysmal dysarthria caused by multiple sclerosis: a case report. J Neurol (2011) 258(7):1349–50. 10.1007/s00415-011-5901-8 21264473

[15] Weier K Till C Fonov V Yeh EA Arnold DL Banwell B Contribution of the cerebellum to cognitive performance in children and adolescents with multiple sclerosis. Mult Scler (2016) 22(5):599–607. 10.1177/1352458515595132 26203072

[16] Tedesco AM Chiricozzi FR Clausi S Lupo M Molinari M Leggio MG. The cerebellar cognitive profile. Brain (2011) 134(Pt 12):3672–86. 10.1093/brain/awr266 22036960

[17] Weier K Penner IK Magon S Amann M Naegelin Y Andelova M Cerebellar abnormalities contribute to disability including cognitive impairment in multiple sclerosis. PLoS One (2014) 9(1):e86916. 10.1371/journal.pone.0086916 24466290 PMC3899307

[18] Peterson JW Bö L Mörk S Chang A Trapp BD. Transected neurites, apoptotic neurons, and reduced inflammation in cortical multiple sclerosis lesions. Ann Neurol (2001) 50(3):389–400. 10.1002/ana.1123 11558796

[19] Lucchinetti CF Popescu BF Bunyan RF Moll NM Roemer SF Lassmann H Inflammatory cortical demyelination in early multiple sclerosis. N Engl J Med (2011) 365(23):2188–97. 10.1056/NEJMoa1100648 22150037 PMC3282172

[20] Kutzelnigg A Faber-Rod JC Bauer J Lucchinetti CF Sorensen PS Laursen H Widespread demyelination in the cerebellar cortex in multiple sclerosis. Brain Pathol (2007) 17(1):38–44. 10.1111/j.1750-3639.2006.00041.x 17493036 PMC8095596

[21] Mainero C Louapre C Meningeal inflammation in multiple sclerosis: the key to the origin of cortical lesions? Neurology (2015) 85(1):12–3. 10.1212/WNL.0000000000001586 25888561

[22] Redondo J Kemp K Hares K Rice C Scolding N Wilkins A. Purkinje cell pathology and loss in multiple sclerosis cerebellum. Brain Pathol (2015) 25(6):692–700. 10.1111/bpa.12230 25411024 PMC4780274

[23] Trapp BD Peterson J Ransohoff RM Rudick R Mörk S Bö L. Axonal transection in the lesions of multiple sclerosis. N Engl J Med (1998) 338(5):278–85. 10.1056/NEJM199801293380502 9445407

[24] Kemp K Redondo J Hares K Rice C Scolding N Wilkins A. Oxidative injury in multiple sclerosis cerebellar grey matter. Brain Res (2016) 1642:452–60. 10.1016/j.brainres.2016.04.027 27086975

[25] Black JA Dib-Hajj S Baker D Newcombe J Cuzner ML Waxman SG. Sensory neuron-specific sodium channel SNS is abnormally expressed in the brains of mice with experimental allergic encephalomyelitis and humans with multiple sclerosis. Proc Natl Acad Sci U S A (2000) 97(21):11598–602. 10.1073/pnas.97.21.11598 11027357 PMC17246

[26] Craner MJ Lo AC Black JA Baker D Newcombe J Cuzner ML Annexin II/p11 is up-regulated in Purkinje cells in EAE and MS. Neuroreport (2003) 14(4):555–8. 10.1097/00001756-200303240-00005 12657884

[27] Renganathan M Gelderblom M Black JA Waxman SG. Expression of Nav1.8 sodium channels perturbs the firing patterns of cerebellar Purkinje cells. Brain Res (2003) 959(2):235–42. 10.1016/S0006-8993(02)03750-2 12493611

[28] Shields SD Cheng X Gasser A Saab CY Tyrrell L Eastman EM A channelopathy contributes to cerebellar dysfunction in a model of multiple sclerosis. Ann Neurol (2012) 71(2):186–94. 10.1002/ana.22665 22367990

[29] Kemp K Gray E Wilkins A Scolding N. Purkinje cell fusion and binucleate heterokaryon formation in multiple sclerosis cerebellum. Brain (2012) 135(Pt 10):2962–72. 10.1093/brain/aws226 22975392

[30] Kemp K Wilkins A Scolding N. Cell fusion in the brain: two cells forward, one cell back. Acta Neuropathol (2014) 128(5):629–38. 10.1007/s00401-014-1303-1 24899142 PMC4201757

[31] Kurtzke JF. Rating neurologic impairment in multiple sclerosis: an expanded disability status scale (EDSS). Neurology (1983) 33(11):1444–52. 10.1212/WNL.33.11.1444 6685237

[32] Fischer JS Rudick RA Cutter GR Reingold SC The multiple sclerosis functional composite measure (MSFC): an integrated approach to MS clinical outcome assessment. National MS Society Clinical Outcomes Assessment Task Force. Mult Scler (1999) 5(4):244–50. 10.1191/135245899678846168 10467383

[33] Calabrese M Mattisi I Rinaldi F Favaretto A Atzori M Bernardi V Magnetic resonance evidence of cerebellar cortical pathology in multiple sclerosis. J Neurol Neurosurg Psychiatry (2010) 81(4):401–4. 10.1136/jnnp.2009.177733 19965849

[34] Anderson VM Fisniku LK Altmann DR Thompson AJ Miller DH. MRI measures show significant cerebellar gray matter volume loss in multiple sclerosis and are associated with cerebellar dysfunction. Mult Scler (2009) 15(7):811–7. 10.1177/1352458508101934 19465449

[35] Polman CH Reingold SC Banwell B Clanet M Cohen JA Filippi M Diagnostic criteria for multiple sclerosis: 2010 revisions to the McDonald criteria. Ann Neurol (2011) 69(2):292–302. 10.1002/ana.22366 21387374 PMC3084507

[36] Anderson VM Wheeler-Kingshott CA Abdel-Aziz K Miller DH Toosy A Thompson AJ A comprehensive assessment of cerebellar damage in multiple sclerosis using diffusion tractography and volumetric analysis. Mult Scler (2011) 17(9):1079–87. 10.1177/1352458511403528 21511688 PMC3281565

[37] Preziosa P Rocca MA Mesaros S Pagani E Drulovic J Stosic-Opincal T Relationship between damage to the cerebellar peduncles and clinical disability in multiple sclerosis. Radiology (2014) 271(3):822–30. 10.1148/radiol.13132142 24555637

[38] Prosperini L Sbardella E Raz E Cercignani M Tona F Bozzali M Multiple sclerosis: white and gray matter damage associated with balance deficit detected at static posturography. Radiology (2013) 268(1):181–9. 10.1148/radiol.13121695 23533287

[39] Davie CA Barker GJ Webb S Tofts PS Thompson AJ Harding AE Persistent functional deficit in multiple sclerosis and autosomal dominant cerebellar ataxia is associated with axon loss. Brain (1995) 118(Pt 6):1583–92. 10.1093/brain/118.6.1583 8595487

[40] Moroso A Ruet A Deloire M Lamargue-Hamel D Cubizolle S Charré-Morin J Cerebellar assessment in early multiple sclerosis. Cerebellum (2017) 16(2):607–11. 10.1007/s12311-016-0831-8 27815857

[41] Kuerten S Kostova-Bales DA Frenzel LP Tigno JT Tary-Lehmann M Angelov DN MP4- and MOG:35-55-induced EAE in C57BL/6 mice differentially targets brain, spinal cord and cerebellum. J Neuroimmunol (2007) 189(1–2):31–40. 10.1016/j.jneuroim.2007.06.016 17655940 PMC2083209

[42] Mandolesi G Grasselli G Musella A Gentile A Musumeci G Sepman H GABAergic signaling and connectivity on Purkinje cells are impaired in experimental autoimmune encephalomyelitis. Neurobiol Dis (2012) 46(2):414–24. 10.1016/j.nbd.2012.02.005 22349452

[43] MacKenzie-Graham A Tiwari-Woodruff SK Sharma G Aguilar C Vo KT Strickland LV Purkinje cell loss in experimental autoimmune encephalomyelitis. Neuroimage (2009) 48(4):637–51. 10.1016/j.neuroimage.2009.06.073 19589388 PMC2754586

[44] Wilkins A Kondo Y Song J Liu S Compston A Black JA Slowly progressive axonal degeneration in a rat model of chronic, nonimmune-mediated demyelination. J Neuropathol Exp Neurol (2010) 69(12):1256–69. 10.1097/NEN.0b013e3181ffc317 21107138

[45] Kapoor R Furby J Hayton T Smith KJ Altmann DR Brenner R Lamotrigine for neuroprotection in secondary progressive multiple sclerosis: a randomised, double-blind, placebo-controlled, parallel-group trial. Lancet Neurol (2010) 9(7):681–8. 10.1016/S1474-4422(10)70131-9 20621711

[46] Kemp K Gordon D Wraith DC Mallam E Hartfield E Uney J Fusion between human mesenchymal stem cells and rodent cerebellar Purkinje cells. Neuropathol Appl Neurobiol (2011) 37(2):166–78. 10.1111/j.1365-2990.2010.01122.x 20819172 PMC4150530

[47] Stewart T Spelman T Havrdova E Horakova D Trojano M Izquierdo G Contribution of different relapse phenotypes to disability in multiple sclerosis. Mult Scler (2017) 23(2):266–76. 10.1177/1352458516643392 27055805

[48] Fox EJ Wynn D Coles AJ Palmer J Margolin DH CAMMS223 Investigators. Alemtuzumab improves neurological functional systems in treatment-naive relapsing-remitting multiple sclerosis patients. J Neurol Sci (2016) 363:188–94. 10.1016/j.jns.2016.02.025 27000249

[49] Armutlu K Karabudak R Nurlu G. Physiotherapy approaches in the treatment of ataxic multiple sclerosis: a pilot study. Neurorehabil Neural Repair (2001) 15(3):203–11. 10.1177/154596830101500308 11944742

[50] Freeman JA Gear M Pauli A Cowan P Finnigan C Hunter H The effect of core stability training on balance and mobility in ambulant individuals with multiple sclerosis: a multi-centre series of single case studies. Mult Scler (2010) 16(11):1377–84. 10.1177/1352458510378126 20699285

[51] Straudi S Martinuzzi C Pavarelli C Sabbagh Charabati A Benedetti MG Foti C A task-oriented circuit training in multiple sclerosis: a feasibility study. BMC Neurol (2014) 14:124. 10.1186/1471-2377-14-124 24906545 PMC4059088

[52] Salcı Y Fil A Armutlu K Yildiz FG Kurne A Aksoy S Effects of different exercise modalities on ataxia in multiple sclerosis patients: a randomized controlled study. Disabil Rehabil (2016):1–7. 10.1080/09638288.2016.1236411 27794631

[53] Mills RJ Yap L Young CA. Treatment for ataxia in multiple sclerosis. Cochrane Database Syst Rev (2007) (1). 10.1002/14651858.CD005029.pub2 17253537

[54] Sabra AF Hallett M Sudarsky L Mullally W. Treatment of action tremor in multiple sclerosis with isoniazid. Neurology (1982) 32(8):912–3. 10.1212/WNL.32.8.912 7201590

[55] Duquette P Pleines J du Souich P. Isoniazid for tremor in multiple sclerosis: a controlled trial. Neurology (1985) 35(12):1772–5. 10.1212/WNL.35.12.1772 3906430

[56] Morrow J McDowell H Ritchie C Patterson V Isoniazid and action tremor in multiple sclerosis. J Neurol Neurosurg Psychiatry (1985) 48(3):282–3. 10.1136/jnnp.48.3.282 3981201 PMC1028268

[57] Francis DA Grundy D Heron JR. The response to isoniazid of action tremor in multiple sclerosis and its assessment using polarised light goniometry. J Neurol Neurosurg Psychiatry (1986) 49(1):87–9. 10.1136/jnnp.49.1.87 3958735 PMC1028653

[58] Koller WC. Pharmacologic trials in the treatment of cerebellar tremor. Arch Neurol (1984) 41(3):280–1. 10.1001/archneur.1984.04050150058017 6365047

[59] Feys P D’hooghe MB Nagels G Helsen WF. The effect of levetiracetam on tremor severity and functionality in patients with multiple sclerosis. Mult Scler (2009) 15(3):371–8. 10.1177/1352458508099142 19168602

[60] Zajicek J Fox P Sanders H Wright D Vickery J Nunn A Cannabinoids for treatment of spasticity and other symptoms related to multiple sclerosis (CAMS study): multicentre randomised placebo-controlled trial. Lancet (2003) 362(9395):1517–26. 10.1016/S0140-6736(03)14738-1 14615106

[61] Fox P Bain PG Glickman S Carroll C Zajicek J. The effect of cannabis on tremor in patients with multiple sclerosis. Neurology (2004) 62(7):1105–9. 10.1212/01.WNL.0000118203.67138.3E 15079008

[62] Wade DT Makela P Robson P House H Bateman C. Do cannabis-based medicinal extracts have general or specific effects on symptoms in multiple sclerosis? A double-blind, randomized, placebo-controlled study on 160 patients. Mult Scler (2004) 10(4):434–41. 10.1191/1352458504ms1082oa 15327042

[63] Twomey JA Espir ML. Paroxysmal symptoms as the first manifestations of multiple sclerosis. J Neurol Neurosurg Psychiatry (1980) 43(4):296–304. 10.1136/jnnp.43.4.296 7373330 PMC490532

[64] Yap L Kouyialis A Varma TR. Stereotactic neurosurgery for disabling tremor in multiple sclerosis: thalamotomy or deep brain stimulation? Br J Neurosurg (2007) 21(4):349–54. 10.1080/02688690701544002 17676453

[65] Timmermann L Deuschl G Fogel W Hilker R Kupsch A Lange M [Deep brain stimulation for tremor in multiple sclerosis: consensus recommendations of the German Deep Brain Stimulation Association]. Nervenarzt (2009) 80(6):673–7. 10.1007/s00115-009-2697-1 19471902

[66] Schuurman PR Bosch DA Bossuyt PM Bonsel GJ van Someren EJ de Bie RM A comparison of continuous thalamic stimulation and thalamotomy for suppression of severe tremor. N Engl J Med (2000) 342(7):461–8. 10.1056/NEJM200002173420703 10675426

[67] Hassan A Ahlskog JE Rodriguez M Matsumoto JY. Surgical therapy for multiple sclerosis tremor: a 12-year follow-up study. Eur J Neurol (2012) 19(5):764–8. 10.1111/j.1468-1331.2011.03626.x 22248187

[68] Zakaria R Vajramani G Westmoreland L Fletcher N Eldridge P Alusi S Tremor reduction and quality of life after deep brain stimulation for multiple sclerosis-associated tremor. Acta Neurochir (Wien) (2013) 155(12):2359–64. 10.1007/s00701-013-1848-0 23975649

